# Structural basis for antigen recognition by methylated lysine–specific antibodies

**DOI:** 10.1074/jbc.RA120.015996

**Published:** 2020-12-17

**Authors:** Misaki Ishii, Makoto Nakakido, Jose M.M. Caaveiro, Daisuke Kuroda, C.J. Okumura, Toshiaki Maruyama, Kevin Entzminger, Kouhei Tsumoto

**Affiliations:** 1 Department of Bioengineering, School of Engineering, The University of Tokyo, Tokyo, Japan; 2 Department of Chemistry and Biotechnology, School of Engineering, The University of Tokyo, Tokyo, Japan; 3 Laboratory of Global Healthcare, Graduate School of Pharmaceutical Sciences, Kyushu University, Fukuoka, Japan; 4 Medical Device Development and Regulation Research Center, School of Engineering, The University of Tokyo, Tokyo, Japan; 5 Abwiz Bio Inc, San Diego, California, USA; 6 Laboratory of Medical Proteomics, The Institute of Medical Science, The University of Tokyo, Tokyo, Japan

**Keywords:** protein methylation, antibody, antigen recognition, biophysics, crystal structure, BSA, bovine serum albumin, HRP, horseradish peroxidase, KLH, keyhole limpet hemocyanin, mAb, monoclonal antibody, MD, molecular dynamics, PTM, posttranslational modifications, SPR, surface plasmon resonance

## Abstract

Proteins are modulated by a variety of posttranslational modifications including methylation. Despite its importance, the majority of protein methylation modifications discovered by mass spectrometric analyses are functionally uncharacterized, partly owing to the difficulty in obtaining reliable methylsite-specific antibodies. To elucidate how functional methylsite-specific antibodies recognize the antigens and lead to the development of a novel method to create such antibodies, we use an immunized library paired with phage display to create rabbit monoclonal antibodies recognizing trimethylated Lys260 of MAP3K2 as a representative substrate. We isolated several methylsite-specific antibodies that contained unique complementarity determining region sequence. We characterized the mode of antigen recognition by each of these antibodies using structural and biophysical analyses, revealing the molecular details, such as binding affinity toward methylated/nonmethylated antigens and structural motif that is responsible for recognition of the methylated lysine residue, by which each antibody recognized the target antigen. In addition, the comparison with the results of Western blotting analysis suggests a critical antigen recognition mode to generate cross-reactivity to protein and peptide antigen of the antibodies. Computational simulations effectively recapitulated our biophysical data, capturing the antibodies of differing affinity and specificity. Our exhaustive characterization provides molecular architectures of functional methylsite-specific antibodies and thus should contribute to the development of a general method to generate functional methylsite-specific antibodies by *de novo* design.

Proteins are modified by a variety of posttranslational modifications (PTM) including phosphorylation, acetylation, glycosylation, and methylation, which are known to play key roles in modulating protein function. Protein methylation is one of the most important histone modifications, affecting changes in gene transcription (1, 2). Recently, numerous methylation sites in nonhistone proteins have been discovered, and methylation has been reported to play a role in both fundamental biological processes and disease states such as cancer (3, 4, 5). Nearly 1% of human genes encode methyltransferases (6), highlighting the diversity of ligands and the importance of methylation in maintaining homeostasis. Yet the majority of methyltransferases remain to be functionally characterized. Indeed, although growing numbers of methylation sites in nonhistone proteins have been identified using recently developed mass spectrometry–based techniques (7, 8), the function of specific methylations often remains unknown (4, 9).

Historically, antibodies have played a major role in advancing basic and translational research into PTM. The advent of phosphosite-specific antibodies led to an explosion in phosphorylation research and demonstrated the ubiquity of this protein modification in cellular processes. However, methylsite-specific monoclonal antibodies remain rare to nonexistent (10), and lack of highly specific antibodies often becomes a bottleneck for subsequent functional studies after identifying a new methylation site (11).

For both ease of use and adaptability to currently available techniques, monoclonal antibodies remain ideal tools for the recognition of site-specific PTM. Although several studies have attempted to create artificial receptors that can recognize methylated lysines (12), these cannot be easily employed in basic immunochemical assays such as Western blotting, immunocytochemistry, and immunohistochemistry owing to low affinity and a lack of sequence specificity surrounding the modification site. Indeed, protein methylation is considered one of the most difficult targets to create modification-specific antibodies owing to the minute differences in chemical moieties brought by the addition of -CH_3_ group(s) (10). Selection-based technology (such as phage display) rather than screening-based technologies (such as hybridoma methods) can take advantage of the power of directed evolution to select for antibody clones possessing the desired function, given sufficient selection pressure during iterative rounds of panning.

Typically, a peptide sequence containing the target modification is used for immunization, with subsequent selection from the immune repertoire of the immunized animal to obtain the site-specific antibodies. However, the dominant immune response elicits modification-specific antibodies that only recognize peptide antigen in ELISA, while antibodies that also recognize protein antigen in biochemical applications such as Western blot are less common. In particular, most currently available methylation-specific monoclonal antibodies target histone tails, which are unstructured and thought to behave like peptides (1, 10).

In this study, we generated trimethylated lysine-specific antibodies using a methylated peptide derived from MAP3K2 (Lys260). MAP3K2 is methylated by the oncogenic methyltransferase SMYD3, and methylation of Lys260 has been linked to functional regulation of this kinase (13, 14). The antibody was created using our original technology that pairs rabbit immunization with selection using fully rabbit (not chimeric) Fab-phage display, and this report can be considered the first detailed characterization of an antibody obtained with this technology. Six unique methylsite-specific Fabs were identified by initial peptide ELISA and further analyzed by ELISA, surface plasmon resonance (SPR), and Western blot. Further detailed analysis of the antibody–antigen complex for four Fabs was performed by X-ray crystallography and molecular dynamics simulations. Together with the results of functional analyses, we discuss the characteristics of molecular recognition by functional methylsite-specific antibodies.

## Results

### Generation of methylated lysine–specific antibodies

**Figure 1:**
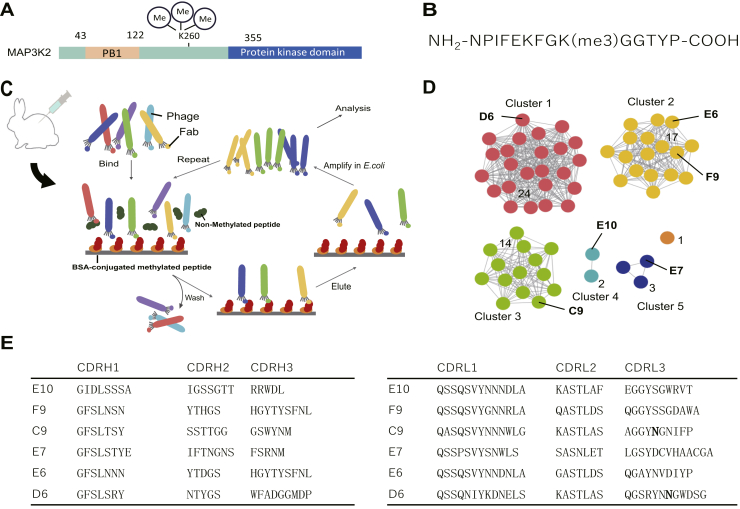
**Acquisition of methylation-specific antibodies using rabbit immunization followed by phage display-based selection.***A*, location of the methylated lysine with domain structures in the MAP3K2 gene. The methylation site is thought to locate in the middle of a large unstructured region. *B*, amino acid sequence of the methylated peptide used for immunization and each experiment. *C*, schematic of antibody acquisition. Rabbits were immunized and Fab–phage display libraries were constructed. Four rounds of biopanning were conducted to amplify antigen-specific Fabs. *D*, the result of sequence-based clustering. Each *circle* indicates a unique Fab clone. The number of clones in each cluster is shown in the middle or to the side of each cluster. *E*, amino acid sequences of CDR loops in picked up clones.

To generate methylsite-specific antibodies, rabbits were immunized with a MAP3K2 peptide sequence surrounding trimethylated Lys260 (Fig. 1, *A*–*B*), N-terminally conjugated to keyhole limpet hemocyanin (KLH). After confirmation of a robust and methylation-specific serum response by ELISA, bone marrow and spleen RNA were extracted and a Fab phagemid library was constructed. Fabs were selected by several rounds of biopanning on bovine serum albumin (BSA)-conjugated methylated peptide antigen, with subtraction of nonspecific binders by including an excess of soluble nonmethylated peptide in solution during the binding step (Fig. 1*C*). Screening of selected Fab clones was carried out by soluble Fab-ELISA (Fig. S1). The absorbance derived from the binding to the BSA-conjugated methylated peptide was compared with that of the unconjugated nonmethylated peptide, and the DNA sequences of methyl-specific clones were analyzed.

The Fab clones were divided into six clusters (Fig. 1*D*) based on the identities of the CDR H3 sequences, and six Fab clones, named E10, F9, C9, E7, E6, and D6, from each cluster that contained more than two unique sequences (clusters 1–5) were chosen for further analysis. The ELISA signal for each clone is summarized in Table S1, and the CDR sequences (based on Chothia definition) of select clones are given in Figure 1*E*. It is notable that despite the Fabs recognizing the same antigen, each of the Fabs characterized in detail possesses a unique CDR L3 sequence of 10 to 13 amino acids in length and a unique CDR H3 sequence (with the exception of two clones sharing identical H3 sequences) of 5, 6, or 9 amino acids in length.

### Binding specificity of each Fab clone for methylated *versus* nonmethylated peptide

**Figure 2:**
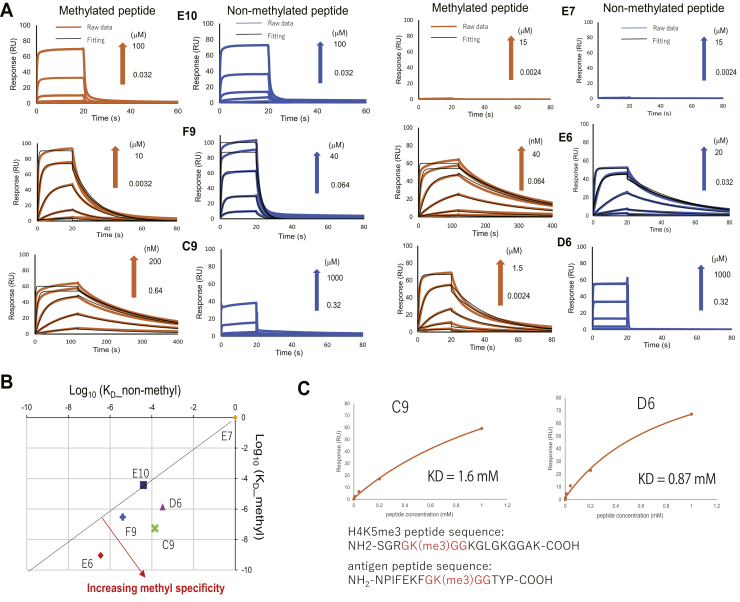
**Surface plasmon resonance (SPR) analysis of Fab–peptide binding.***A*, SPR response curves toward both methylated and nonmethylated peptides for each clone. *B*, Fab affinity plot. Vertical and horizontal axes indicate the affinity of each Fab clone toward methylated and nonmethylated peptides, respectively. Clones with the furthest perpendicular distance from the diagonal line possess the highest peptide specificity. *C*, Affinity curves derived from SPR analysis of the interaction between Fab clones and a histone H4 peptide containing K5 tri-methylation. The C9 and D6 showed detectable binding affinity.

To evaluate the specificity against methylation, we prepared each antibody as a Fab construct, which consists of the antigen-binding domain in antibodies (15), as a purified recombinant protein and determined binding affinity as well as kinetic parameters toward both unconjugated trimethylated and nonmethylated peptides by using surface plasmon resonance (SPR) (Fig. 2*A* and Fig. S2). The *K*_*D*_ values toward methylated and nonmethylated peptides for all the Fabs are plotted in Figure 2*B*, and the binding affinity and kinetic parameters of the interaction are summarized in Table S2.

The result showed that F9, C9, E6, and D6 preferentially bound to the methylated peptide as consistent with Fab ELISA. These clones possessed affinities of 3.0 ×10^2^, 14, 0.90, and 54 nM, respectively, for the methylated peptide. Affinities for the nonmethylated peptides were within the millimolar to 0.3-μM range. It is surprising that E10 showed a similar weak binding to both peptides with 40 μM *K*_*D*_ affinity, contrary to ELISA. It is intriguing that E7 did not show any significant binding response in SPR, although ELISA indicates a strong binding activity to the methylated peptide. This is presumably a result of the different peptide presentations in the two assays; ELISA used immobilized, BSA-conjugated peptide antigen, and SPR used soluble free peptide antigen (with immobilized Fab). To examine this hypothesis, we prepared the methylated peptide conjugated with another carrier protein human serum albumin and conducted ELISA analysis. The result showing that E7 bound to the conjugated peptide (Fig. S3) would support the above hypothesis that E7 recognized not solely the peptide sequence but the local structure induced by conjugation with carrier proteins.

Furthermore, as MAP3K2 Lys260 can also be mono- and di-methylated (16), we conducted SPR analysis using mono- and di-methylated peptides to evaluate the specificity of each antibody toward the methylation state of the peptide. The result showed that the affinity increased with greater methylation degree, although this dependency was not identical among the antibodies (Table S3). These results suggest that, although the antibodies also recognize the mono- and di-methylated antigen, they have some specificity (especially for C9 and D6) toward the degree of methylation.

To assess the sequence specificity for the Fabs possessing high affinity to the methylated peptide, F9, C9, E6, and D6 were tested by SPR using a histone H4 peptide containing K5 trimethylation, which possess three consensus amino acid sequences surrounding the methylation site with MAP3K2 (GK(me3)GG). Although C9 and D6 showed detectable binding to the H4 peptide, the affinity was significantly lower than that toward the target peptide (Fig. 2*C*). In addition, C9 and D6 showed essentially no affinity (approximately millimolar level) to a peptide derived from histone H3 containing a trimethylated K27, which possess different amino acids sequences surrounding the trimethylated lysine (Fig. S4).

These results suggest that these Fabs are not only methylated lysine specific but are also methylsite specific, as they have a strong specificity toward methylated lysine in the context of MAP3K2 surrounding amino acids.

### Recognition of the antigen in the context of the full-length protein

**Figure 3:**
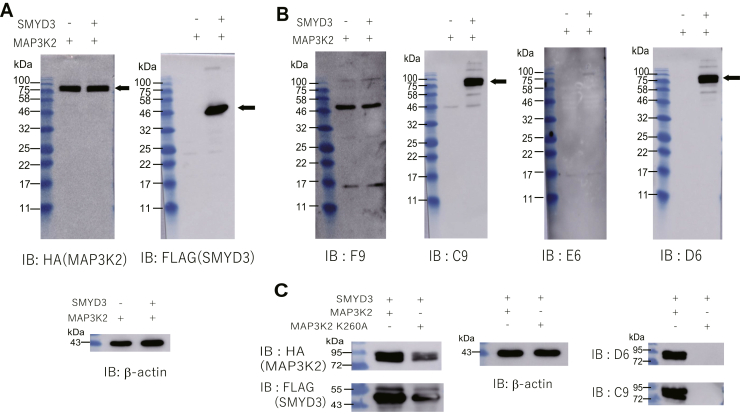
**Western blotting analysis.***A*, confirmation of expression of MAP3K2 and SMYD2. The cell lysates from HEK293 cells cotransfected with HA-tagged MAP3K2 and FLAG-tagged SMYD3 or mock expression vectors were separated by SDS-PAGE, blotted to membranes, and stained with anti-HA, anti-FLAG tag, or anti β-actin antibodies. *B*, western blotting using each IgG. The membranes were stained with each IgG followed by incubation with an HRP-conjugated anti-rabbit IgG antibody. F9 and E6 lack clear band signal derived from binding to the protein antigen, while C9 and D6 show strong binding only in the presence of SMYD3. *C*, western blotting using the MAP3K2 K260A mutant. The mutation significantly diminished the corresponding band.

We next sought to test whether these Fabs recognize methylated protein antigen in the context of other cellular proteins. We converted the clones to rabbit IgG format for Western blot analysis. HEK293 cells were transfected with a MAP3K2 expression vector together with either a mock or SMYD3 expression vector, as SMYD3 upregulation has been shown to methylate Lys260 of MAP3K2 (13, 14). Both MAP3K2 and SMYD3 expressions were confirmed by analysis of HA or FLAG tags, respectively (Fig. 3*A*). Each IgG clone was then tested by Western blot (Fig. 3*B*). Two off-target bands appeared on staining with F9, neither of which corresponds to the expected size of MAP3K2 and the intensity of which is not dependent on SMYD3 expression. This result indicates that F9 does not appear to recognize protein antigen. On the contrary, the band corresponding to MAP3K2 was clearly shown by staining with C9 and D6 in a SMYD3 expression-dependent manner. Although some bands, which might be derived from other trimethylated proteins that possess similar amino acids surrounding methylated lysine, were also observed in a SMYD3 expression-dependent manner, the band derived from MAP3K2 was dominant, guaranteeing the specificity of the antibodies. Furthermore, to verify that these antibodies indeed recognized the methylated Lys260, detection using the MAP3K2 K260A mutant was tested. The corresponding band was significantly diminished by the mutation for both C9 and D6 (Fig. 3*C*), demonstrating that these antibodies specifically recognize methylated Lys260 residue. It is intriguing that E6 staining only showed a faint band despite possessing the highest affinity among the Fabs toward the methylated peptide. These results indicate that antigen affinity alone is not predictive of antibody behavior in other applications such as Western blotting, especially when the application involves full-length protein antigen.

### Structural analysis of Fab–peptide complexes

**Figure 4:**
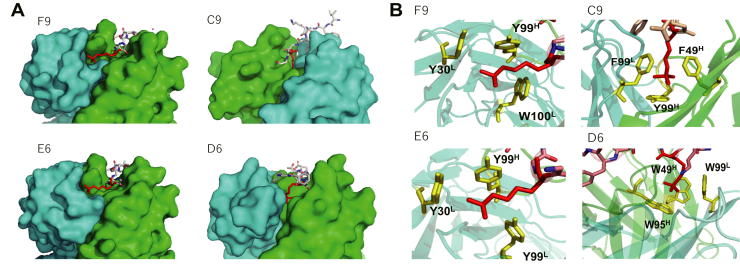
**Crystal structures of Fab–peptide complexes.***A*, overview of the peptide binding region for each Fab is shown. Heavy and light chains are colored *green* and *cyan*, respectively; the C9 structure is shown in a different orientation with respect to that of other Fabs. The methylated lysine residue in the peptide is highlighted *red*. F9 and E6 show a binding mode in which the methylated lysine pointed toward the light chain, while the methylated lysine is deeply buried in the interchain interface for both C9 and D6. *B*, enlarged view focusing on the methylated lysine residues and the surrounding aromatic cage are shown. The methylated lysine residues (*red*) are caged by aromatic residues (*yellow*) in each of the Fab structures.

To gain further insight into how the antibodies recognize the antigen, we determined the crystal structures of each Fab–methylated peptide complex. Omit electron density maps of the peptides indicate with a high degree of confidence that these peptides are bound to the antibodies in the crystal (Fig. S5). Antibodies F9 and E6 engage the peptide predominantly using their heavy chain, with the methylated lysine pointing toward the light chain (Fig. 4*A*). These similar binding modes could be expected, as the heavy chain CDRs of F9 and E6 differ by only a single amino acid in CDRs H1 and H2 (Fig. 1*E*). On the other hand, the methylated lysine residue was deeply buried in the interface between the heavy and light chains in C9 and in D6, both of which recognized the protein antigen in Western blotting. Indeed, the calculated accessible surface area of the methylated lysine residue side chain in each complex (F9, 45.3 Å^2^; C9, 3.8 Å^2^; E6, 34.0 Å^2^; and D6, 0.0 Å^2^) clearly demonstrates the nearly complete penetration of the methylated lysine residue into the interchain cleft for C9 and D6.

Of importance, both the N-terminal and C-terminal portions of the peptide were not observed in the complex suggesting that each terminus is disordered. This binding mode may be compatible with antigen recognition in the context of the intact protein, as would occur in Western blotting. Meanwhile, the full C terminus of the peptide was observed in structures of the complex in other Fabs.

By studying the local environment surrounding the methylated lysine residues, we found that the four antibody clones examined recognize the methylated lysine residue by using several aromatic residues in a cooperative manner (Fig. 4*B*). The pockets formed with the aromatic residues, termed “aromatic cage,” are also observed in reader proteins working in the recognition of histone modifications and an antimethylated histone antibody (10), which specifically recognize methylated lysine residues (17). These structures strongly suggest that these Fabs acquired their specificity toward methylation by using the same structural principles. Considering that each Fab uses different combinations of aromatic residues, *i.e.*, F9 uses two Tyr and one Trp, C9 uses two Phe and one Tyr, E6 uses three Tyr, and D6 uses three Trp, the combination of aromatic residue is not likely to be a fundamental for the recognition of methylated lysine residue, instead indicating the general importance of the CH–pi interaction (18) between the aromatic residues and methyl groups in the methylated lysine.

Looking at the entire peptide in the complexes, each Fab also made hydrogen bonds as well as salt bridges with other parts of the peptide. All the hydrogen bonds and salt bridges, calculated by PDBePISA (19), are summarized in Supplementary Note 1. F9 and E6 each engaged in 17 hydrogen bonds and 4 salt bridges with the methylated peptide (Fig. S6). On the other hand, C9 and D6 each made seven hydrogen bonds with the peptide (Fig. S6). The fewer hydrogen bonds observed in C9 and D6 compared with F9 and E6, together with the comparable affinity ranges among the Fabs, also suggests the large contribution that deeply located aromatic cages play in peptide binding.

Of importance, F9 and E6 engaged in several hydrogen bonds as well as salt bridges with the peptide’s C-terminal carboxyl group, which may explain why these Fabs cannot recognize the full-length protein. To confirm the contribution of these noncovalent bonds with the C-terminal carboxyl group, we conducted SPR analysis using a methylated peptide in which the C terminus was amidated (Fig. S7). The results show that the binding response of the amidated peptide was nearly abolished for F9 and E6, whereas in contrast C9 and D6 recognized the amidated peptide with comparable affinity with that of the nonamidated peptide. This demonstrates that the major reason that F9 and E6 do not recognize protein antigen is their bonding to the C-terminal carboxyl group, which would be incompatible with binding in the context of the full protein sequence.

### Molecular dynamics simulations of holo- and apo-Fab

To further characterize the recognition of the methylated lysine residue by the aromatic cages and to explore the possibility of *in silico* prediction of specificity toward methylation for each antibody, we conducted multiple 400-ns molecular dynamics simulations for each Fab–peptide complex.

**Figure 5:**
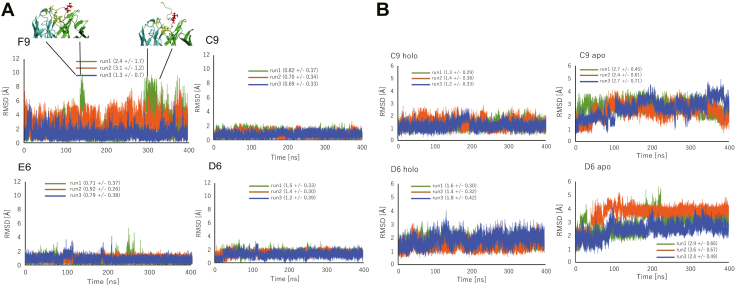
**MD simulation analyses of Fab.***A*, RMSD values of methylated lysine NZ atom in each simulation were plotted against simulation time for each Fab clone. F9 showed highly dynamic variability, while C9, E6, and D6 effectively captured the peptide during the length of the simulation; structures of the F9 complex at time points where the methylated lysine residue was thrown out were overlayed with the plot. *B*, RMSD values of the aromatic cages in C9 and D6 in the presence (holo) or absence (apo) of the antigen peptide were plotted against simulation time. Increase of the RMSD observed in C9 and D6 apo structures may suggest the collapse of the aromatic cage.

First, we calculated the RMSD values of the NZ atoms of the methylated lysine after superposing the side-chain atoms of aromatic residues in each structure. The RMSD plots indicate that the methylated lysine residue was stably bound by the aromatic cage in C9, E6, and D6, whereas it dynamically moved in the F9 complex (Fig. 5*A*). Of importance, the F9 structure showed that the methylated lysine was completely thrown out from the cage at several time points during the simulations, and the antibody retained the peptide only through interaction with the C terminus (Fig. S8). This exclusion of the methylated lysine residue from the aromatic cage was also confirmed by the calculation of the solvent accessible surface area of the methylated lysine residue (Fig. S9). This result appears to be concordant with the SPR results, which showed that C9, E6, and D6 have higher preference toward methylated peptide compared with F9, indicating that our simulations appropriately recaptures antigen recognition observed in the *in vitro* experiments.

We subsequently evaluated the stability of the aromatic cage formation in C9 andD6, both of which recognized protein antigen, to assess if the cages stably form regardless of the antigen. We calculated RMSD values of the all the carbon and nitrogen atoms of the three aromatic residues that form the cages after superposing all the Cα atoms of each complex for both the Fab–methylated peptide complex and apo-Fabs, which were computationally generated based on the crystal structures of the complexes (Fig. 5*B*). The results showing large fluctuation of RMSD values in the apo structures indicate that the relative position of aromatic residues to each other continuously changed; *i.e.*, the cage collapsed in the absence of peptide antigen, suggesting that the aromatic cages would stably form only in the presence of the antigen even in the C9 and D6 antibodies.

## Discussion

In the present study, we created antibodies that recognize the peptide antigen in a methylation-dependent manner by using rabbit immunization followed by antibody library construction and phage display-based selection. This is the first report of detailed structural characterization of antibodies elicited by the use of this novel technique that pairs rabbit immunization with phage display. It is worth noting that, to the best of our knowledge, this is also the first study to characterize the structural and biophysical basis for recognition of a protein antigen by a series of methylation-specific antibodies.

Rabbit monoclonal antibodies (mAbs) generally possess higher affinity (picomolar *versus* nanomolar KD) over their mouse and human counterparts (20) owing to extensive somatic hypermutation and even somatic gene conversion, a mechanism absent in humans and mice (21, 22). Owing to an increased CDR3 length and sequence diversity, recombinant rabbit MAbs have been shown to provide high-quality detection for difficult epitopes including PTM such as phosphorylation and methylation, especially when MAbs from other species had failed (23). However, traditional rabbit mAb platforms rely on hybridoma technology or B cell cloning to generate lead candidates and these systems suffer from limited library sizes (10^4^–10^6^, compared with 10^10^–10^11^ in phage display). Also, clone recovery by single cell PCR can be inefficient, causing loss of important lead candidates. Alternatively, historical rabbit phage display systems have failed to compensate for the unique disulfide architecture of rabbit Fabs that can cause expression problems in *Escherichia. coli*, where the most commonly used Ck1 chain possesses an additional disulfide bond that is not found in mouse or human kappa chains. This led to the development of suboptimal systems that are limited to the underutilized Ck2 chain and thus do not use the full rabbit immune repertoire. To overcome these challenges, we used a patented library construction method paired with an optimized proprietary Fab–phage display vector (containing a hairpin structure to prevent transcription by leaky lac-promoter and strictly controlling the production of Fabs in native state) that allows for display of fully rabbit Fab in the natural form without needing to rely on chimeric alternatives. Rabbit antibody genes are amplified using our method, which engineers second strand cDNA to append a matching nucleotide sequence to both 5’ and 3’ ends, allowing for antibody gene amplification using a single non–gene-specific primer (US 9,890,414). Unbiased, robust amplification creates high-quality libraries that preserve the natural frequency of the antibody genes isolated from the immunized rabbit. Phage panning also allows for unique selection conditions to be used, such as removal of cross-reactive clones to related but off-target antigens by subtraction with a soluble competitor.

Although we selected clones that show excellent specificity against methylation in Fab-ELISA, our biophysical analyses of the interactions between peptide and antibodies using recombinant Fab proteins showed differing specificities for the Fabs. As we used KLH- or BSA-conjugated methylated peptide for immunization, phage-based selection, and Fab ELISA steps, this discrepancy may be caused by local structural changes brought by conjugation. In addition, our results showed that recognition of the peptide C terminus by the antibodies precludes cross-reactivity to protein antigen. Immunogen peptide formats including choice of conjugation carriers and C-terminal modifications during each step should be carefully considered to generate functional antibodies that cross-react to protein antigen.

**Figure 6:**
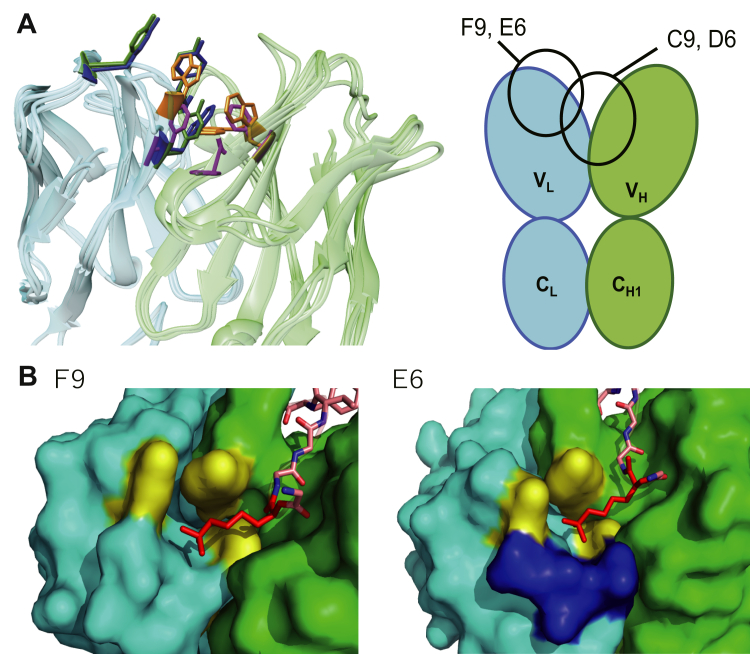
**Toward antibody improvement and design.***A*, all Fab structures are superimposed, with highlighted aromatic residues composing aromatic cages shown. The illustration shows the relative positioning of the aromatic cages. *B*, enlarged view of the methylated lysine (*red*) in the complex structures of F9 and E6. The residues composing aromatic cages (*yellow*) and the aspartic acid residue (*blue*) located adjacent to the aromatic cage in E6 are highlighted.

Our Western blotting and structural analyses suggest that appropriate positioning of the aromatic cage would be a critical factor to promote cross-reactivity to both peptide and protein antigen. Locating an aromatic cage deep within the antibody enables strong grasping of the methylated lysine, generating enough affinity toward both peptide/protein antigens without requiring additional intensive interactions. The superposed structures of all Fabs, including the aromatic cage side chains are shown in Figure 6*A*. Despite using different amino acids, F9 and E6 share good overlap in aromatic cage positioning, as do C9 and D6 separately. Randomization of adjacent residues while retaining the fixed aromatic cage residues could be a promising strategy to generate a library of methylation-specific antibodies to create new sequence specificities. A similar general approach to create PTM-specific antibodies has been proposed for an anti-phosphorylation antibody library (24).

F9 and E6 share a high degree of sequence identity, excluding CDR-L3, and almost identical hydrogen bonds and salt bridges with the peptide. Yet, E6 showed a nearly 300 times higher affinity toward the methylated peptide compared with F9. Looking at the E6 structure, a key aspartic acid residue is located near the aromatic cage (Fig. 6*B*). This negatively charged residue appears to electrostatically attract the methylated lysine and thereby enhance the affinity. Our SPR results show a >100-fold difference in association rate constant between F9 and E6 (Table S2), also supporting the importance of this electrostatic effect.

Finally, we discuss a possibility of rational design of methylation-specific antibodies. Our molecular dynamics (MD) simulation succeeded in recapturing methylation-dependent antigen recognition by antibodies using the aromatic cages. Given that the simulation accurately evaluated the specificity difference between F9 and E6, even though these two antibodies have high sequence identity including the residues forming the aromatic cage, the specificities of designed antibodies toward methylated antigens may be predictable without experiments. Prediction of binding specificity could be a first step in rational engineering of functional protein. In this context, the computer-based prediction would dramatically accelerate the evaluation process of designed antibody and thereby contribute to the rational design of the antibodies.

Although further studies will be required to establish the design strategy, our exhaustive characterization of methylation-specific antibodies should contribute to the development of a general method to generate functional methylsite-specific antibodies.

## Experimental procedures

### Peptide synthesis

All the peptides used in this study were manufactured by SCRUM Inc (Tokyo, Japan), and correct synthesis was confirmed by MS analysis. For immunization and Fab screening, the synthesized peptides were conjugated to carrier protein using N-(6-maleimidocaproyloxy) succinimide. Peptide sequences used in this study are as follows. Methylated MAP3K2 peptide, NPIFEKFGK(me3)GGTYP; MAP3K2 peptide, NPIFEKFGKGGTYP; Histone H4 methylated peptide, SGRGK(me3)GGKGLGKGGAK; Histone H3 methylated peptide, QLATKAARK(me3)SAPATG.

### Rabbit immunization

Two New Zealand white rabbits were immunized subcutaneously with methylated peptide-KLH conjugate four times at 2-week intervals at Prosci Inc (Poway, California). On the first day, the rabbits were immunized with 200 μg of peptide-KLH in PBS with complete Freund’s adjuvant and the preimmune sera were obtained. The following injections were performed with 100 μg of peptide-KLH in PBS with incomplete Freund’s adjuvant. The sera were obtained 1 week after the final injection and antibody titers were tested by ELISA.

### Library construction and selection

The rabbit that showed the best antibody titer against the methylated peptide *versus* the unmethylated peptide was selected for library construction. Total RNA was obtained from bone marrow and spleen using Tri-reagent (Molecular Research Center, Inc, Cincinnati, OH, USA) according to the manufacturer’s instructions. Messenger RNA was obtained using NucleoTrap mRNA (Macherey-Nagel, Bethlehem, PA, USA) spin columns per manufacturer’s instructions. Then, the libraries were made using the method described in detail in a published patent US 9,890,414. Briefly, first-strand cDNA was synthesized from messenger RNA using oligo dT and PowerScribe MMLV RT (Monserate Biotechnology Group, San Diego, CA, USA). Second-strand cDNA synthesis was performed using Amplitaq polymerase (Applied Biosystems) and single primer amplification was then performed using Advantage 2 polymerase mix (Clontech) and incorporated into Fab–phage display vector and incorporated into Fab–phage display vector in which both heavy and light chains, in natural sequences, are contained in the same vector together with each own signal peptide as the widely used Fab–phagemid vectors (25). Heavy chains were fused to a truncated gene III protein of M13 phage as described in the previous study (26). For the amplification of the libraries in *E. coli*, 10 μg of library DNA was electroporated into XL-Blue cells (Monserate) and phage production was induced with VCS M13 helper phage in the presence of 1 mM IPTG and antibiotics (carbenicillin, tetracycline, and kanamycin) at 30 °C overnight. Phage was precipitated from the bacterial supernatant with PEG/NaCl and resuspended in 1% BSA/PBS. Microtiter wells (Costar 3690) were coated with 50 μl methylated peptide–BSA conjugate at 2 μg/ml in PBS at 4 °C overnight. Wells were washed five times with PBS and filled with 1% BSA/PBS then incubated for 1 h at 37 °C. The blocker was flicked out and 200 μl of round 1 input phage was added to each well and incubated for 1.5 h at 37 °C. After washing three times with PBS, bound phage was eluted, neutralized, and used to infect ER2738 *E. coli* cells. Phage was amplified overnight for the next round with VCS M13 helper phage in the presence of 1 mM IPTG and antibiotics (carbenicillin, tetracycline, and kanamycin) at 30 °C overnight. Subsequent rounds 2 to 4 we performed as above in microtiter wells either in the presence or absence of nonmethylated peptide as a soluble competitor at a final concentration of 2 μg/ml to absorb nonmethylated specific binders.

### Fab screening

For the screening of individual Fab clones, the round 4 output bacterial colonies were grown in 1.2 ml of Super Broth medium containing 50 μg/ml of carbenicillin for 4 h 37 °C (850 rpm). Clones were induced with 1 mM IPTG at OD600 0.5 to 0.7 at 30 °C overnight. Microtiter wells (Costar 3590) were coated with 50 μl methylated peptide–BSA conjugate at 1 μg/ml in PBS, 50 μl nonmethylated peptide at 1 μg/ml in PBS and anti-rabbit Fab’2 to measure the expression level (2 μg/ml in PBS; Jackson Immunoresearch#111-006-047) overnight at 4 °C. The wells were washed three times with PBS and blocked with 100 μl of 1% BSA/PBS at 37 °C for 1 h. The culture plate was spun down, the blocker was replaced with 50 μl Fab sup, and incubated at 37 °C for approximately 2 h. The wells were washed three times with PBS and bound Fab was detected with goat anti-rabbit IgG F(ab’)2 horseradish peroxidase (HRP) conjugate (Pierce 31461) (1:5000 in 1% BSA/PBS) at 37 °C for 1 h. The wells were washed 3× with PBS and developed with 50 μl of tetramethylbenzidine substrate mixture. The reaction was stopped with 50 μl of 2 N sulfuric acid after 20 min, and the plates were read at 450 nm.

### Fab preparation as recombinant proteins

Gene fragments encoding heavy and light chains from each Fab clone were cloned into pcDNA 3.4, an expression vector for mammalian expression system (ThermoFisher Scientific) with Igκ signal peptide sequence. Expi293 cells (ThermoFisher Scientific) were cotransfected with expression vectors of heavy and light chains for each Fab, and the supernatant was collected 4 days after transfection. The supernatant was dialyzed against 20 mM Tris-HCl (pH 8.0), 500 mM NaCl, 5 mM imidazole (biding buffer) and loaded on Ni-NTA resin (QIAGEN) equilibrated with the binding buffer. The resin was washed with 20 mM Tris-HCl (pH 8.0), 500 mM NaCl, 20 mM imidazole (wash buffer) and subsequently The Fabs were eluted by 20 mM Tris-HCl (pH 8.0), 500 mM NaCl, 500 mM imidazole. The eluted Fabs were dialyzed against PBS and further purified by size exclusion chromatography using a HiLoad 26/600 Superdex 75-pg column (GE Healthcare) equilibrated with PBS. For crystallization, Fabs were extensively purified by subsequent anion exchange chromatography using a Resource Q column (GE Healthcare) followed by second size exclusion chromatography using the same column as described above in 20 mM Tris-HCl (pH8.0), 20 mM NaCl. The monomer peak fractions were collected and the purity of each Fab was evaluated by SDS-PAGE followed by Coomassie staining.

### Surface plasmon resonance

The bindings of the peptides to Fabs were analyzed in a Biacore T200 instrument (GE Healthcare). Each Fab was immobilized on the surface of a CM5 sensor chip (GE Healthcare) by the amine-coupling method according to the manufacturer’s instructions. PBS containing 0.005% Tween 20 was used as the running buffer. The kinetic data were obtained by injection of increasing concentrations of peptides into the sensor chip. The data were analyzed with the BIAevaluation software (GE Healthcare), and the binding affinity as well as kinetic parameters were calculated by a global fitting of the curves. When a particular combination of Fabs/peptide showed fast dissociation rate constants, the kinetic parameters were difficult to determine by global fitting, and thus the binding affinity was determined by steady-state analysis.

### IgG preparation as recombinant proteins

IgG expression vectors were constructed by conjugating the heavy chains to rabbit IgG Fc by using an antibody-expressing positive control vector for IgG expression (ThermoFisher Scientific) as a template. Expi293 cells were cotransfected with expression vectors of heavy and light chains for each IgG, and the supernatant was collected 4 days after transfection. The supernatant was loaded on rProtein A Sepharose Fast Flow (GE Healthcare) equilibrated with PBS. The resin was washed with PBS, and subsequently the IgGs were eluted by 50 mM sodium citrate buffer (pH 3.0). The eluted fractions were immediately neutralized with 2M Tris-HCl (pH 8.0) and further purified by size exclusion chromatography using a HiLoad 26/600 Superdex 200-pg column (GE Healthcare) equilibrated with PBS.

### Western blotting

The gene encoding MAP3K2 was amplified from an expression vector pDONR223-MAP3K2 from William Hahn & David Root (Addgene plasmid # 23454; http://n2t.net/addgene:23454; RRID:Addgene_23454) (27) and cloned into pcDNA3.4 with a HA tag sequence. HEK293 cells were cotransfected with HA-MAP3K2 and mock or FLAG-SMYD3 expression vectors. Cell lysate samples were prepared from HEK293 cells lysed with radioimmunoprecipitation cell lysis buffer (Santa Cruz) supplemented with complete protease inhibitor cocktail (Roche Applied Science). The total amount of proteins was determined using the BCA Protein Assay Kit (ThermoFisher Scientific), and whole-cell lysates were separated by SDS-PAGE and blotted to nitrocellulose membrane. Protein bands were detected by incubating with anti-DDDDK-tag mAb-HRP-DirecT (MBL Lifescience), anti-HA tag pAb-HRP-DirecT (MBL Lifescience), or each IgG followed by the incubation with HRP-conjugated anti-rabbit IgG, HRP-linked antibody (Cell Signaling Technology) at room temperature for 30 min and visualized with enhanced chemiluminescence (GE Healthcare).

### Crystallization, data collection, and refinement

Purified Fabs were mixed with the methylated peptide, followed by concentration using Amicon Ultra MWCO 10000 (Merck Millipore) to 4.3 (F9), 4.3 (C9), 5.3 (E6), and 4.4 mg/ml (D6). The initial crystallization screening was carried out using an Oryx8 protein crystallization robot (Douglas Instruments). Single crystals for each Fab were obtained in a solution of 0.2 M potassium iodide, 20% PEG3350 (F9), 0.2 M magnesium chloride hexahydrate, 20% PEG3350 (C9), 0.1 M potassium chloride, 24% PEG3350 (E6), or 0.2 M calcium chloride, 20% PEG3350 (D6). Suitable crystals were harvested, briefly incubated in mother liquor supplemented with 20% glycerol, and transferred to liquid nitrogen for storage until data collection.

Diffraction data from single crystals obtained as explained above were collected in beamlines BL5A and BL1A of the Photon Factory (Tsukuba, Japan), or beamline BL-26B2 of Spring 8 (Hyogo, Japan) under cryogenic conditions (100 K). Diffraction images were processed with the program MOSFLM and merged and scaled with the program SCALA or AIMLESS (28) of the CCP4 suite (29). The structure of D6 was determined by the molecular replacement method using the coordinates of Fab NVS-1-19-5 (PDB entry code 5M63) (30) with the program PHASER (31). The structure of the other Fabs was also determined by the method of molecular replacement using the coordinates of D6 as a template. The models were refined with the programs REFMAC5 (32) and built manually with COOT (33). Validation was carried out with PROCHECK (34). Data collection and structure refinement statistics are given in Table S3.

### MD simulations

MD simulations of Fab complexes were performed using GROMACS 2016.3 (35) with the CHARMM36m force field and the CMAP correction (36, 37). Using the CHARMM-GUI (38) the Fab structures were solvated with TIP3P water in a rectangular box such that the minimum distance to the edge of the box was 15 Å under periodic boundary conditions. Na and Cl ions were added to neutralize the protein charge, then further ions were added to mimic a salt solution concentration of 0.14 M. Each system was energy minimized for 5000 steps and equilibrated with NVT ensemble at 298 K for 1 ns. Further production run was performed for 400 ns with NPT ensemble and the time step was set to 2 fs throughout the simulations. A simulation was repeated three times for each system, and the snapshots were saved every 10 ps. PyMOL and UCSF Chimera (39) were employed to analyze and visualize the MD trajectories and to render the molecular graphics. To assess the stability of our simulations, we first computed the RMSD of the Cα atoms after superposing the Cα atoms of each complex during the simulations, with reference to the initial structure of the production runs (Fig. S10), suggesting that our simulations were well equilibrated after 10 ns. solvent accessible surface area values as a function of time were calculated by using gmx sasa in GROMACS (40).

## Data availability

The coordinates and structure factors for the structure of antibodies in complex with methylated peptide have been deposited in the PDB under entry codes 6LDV (F9 + peptide), 6LDW (C9 + peptide), 6LDX (E6 + peptide), and 6LDY (D6 + peptide).

All other data are available from the authors upon request. Please send request to Makoto Nakakido, nakakido@protein.t.u-tokyo.ac.jp.

## Conflict of interest

The authors used patented technology WizAmp (US 9,890,414), invented by CJ. O. and T. M. for antibody acquisition.

## Supporting information

Tables S1–S2

Supplementary Note 1
