# Supplementary material for: Structural basis for antigen recognition by methylated lysine–specific antibodies
Source: J Biol Chem. 2020 Dec 17;296:100176. doi: 10.1074/jbc.RA120.015996 (PMC7948472; doi:10.1074/jbc.RA120.015996)
Supplement: Figures S1–S9 [file mmc1.pdf]

Table S1: Absorbance value of ELISA using phage-Fab for selected clones

Clone	Methylated peptide	Non-methylated peptide
E10	4	0.111
F9	1.762	0.103
C9	4	0.057
E7	4	0.065
E6	4	0.119
D6	4	0.063

Table S2: kinetics parameters of Fab / peptides interaction

Clone		K_D (M)	k_{on} ($M^{-1}s^{-1}$)	k_{off} (s^{-1})	Specificity Ratio* (K_D Non-me/K_D Me)
E10	Methylated peptide	$3.7 \pm 0.2 \times 10^{-5}$	N.D	N.D.	1.1
	Non-methylated peptide	$4.0 \pm 0.4 \times 10^{-5}$	N.D	N.D.	
F9	Methylated peptide	$3.0 \pm 0.1 \times 10^{-7}$	$3.3 \pm 0.4 \times 10^{5}$	$9.8 \pm 1.5 \times 10^{-2}$	13.3
	Non-methylated peptide	$4.0 \pm 0.7 \times 10^{-6}$	$8.6 \pm 2.4 \times 10^{4}$	0.33 ± 0.04	
C9	Methylated peptide	$1.4 \pm 0.3 \times 10^{-8}$	$4.8 \pm 0.4 \times 10^{5}$	$6.0 \pm 0.6 \times 10^{-3}$	31428
	Non-methylated peptide	$4.4 \pm 2.5 \times 10^{-4}$	N.D	N.D.	
E7	Methylated peptide	N.D	N.D	N.D.	N.D.
	Non-methylated peptide	N.D	N.D	N.D.	
E6	Methylated peptide	$9.0 \pm 1.6 \times 10^{-10}$	$3.4 \pm 1.2 \times 10^{6}$	$2.9 \pm 0.6 \times 10^{-3}$	389
	Non-methylated peptide	$3.5 \pm 0.2 \times 10^{-7}$	$5.0 \pm 0.4 \times 10^{5}$	0.18 ± 0.01	
D6	Methylated peptide	$5.4 \pm 0.4 \times 10^{-8}$	$1.9 \pm 1.1 \times 10^{6}$	$9.6 \pm 5.2 \times 10^{-2}$	2593
	Non-methylated peptide	$1.4 \pm 0.4 \times 10^{-4}$	N.D	N.D.	

***The specificity ratio represents the fold difference between affinity for the non-methylated vs. methylated peptide, with a higher ratio corresponding to a more methyl-specific clone**

Table S3 Binding affinity of Fab / peptide with different methylation state

Clone	K_D (M)			
	Trimethyl	Dimethyl	Monomethyl	Non-methyl
F9	$3.0 \pm 0.1 \times 10^{-7}$	$6.9 \pm 1.6 \times 10^{-7}$	$2.2 \pm 0.6 \times 10^{-6}$	$4.0 \pm 0.7 \times 10^{-6}$
C9	$1.4 \pm 0.3 \times 10^{-8}$	$8.4 \pm 2.5 \times 10^{-8}$	$2.3 \pm 1.0 \times 10^{-5}$	$4.4 \pm 2.5 \times 10^{-4}$
E6	$9.0 \pm 1.6 \times 10^{-10}$	$8.4 \pm 1.4 \times 10^{-9}$	$3.6 \pm 0.4 \times 10^{-8}$	$3.5 \pm 0.2 \times 10^{-7}$
D6	$5.4 \pm 0.4 \times 10^{-8}$	$2.3 \pm 0.1 \times 10^{-7}$	$2.5 \pm 0.3 \times 10^{-5}$	$1.4 \pm 0.4 \times 10^{-4}$

Table S4: Data collection and refinement statistics.
Statistical values given in parenthesis refer to the highest resolution bin.

Data Collection	C9 + peptide	D6 + peptide	E6 + peptide	F9 + peptide
Beamline	Spring 8 BL-26B2	PF BL-5A	PF BL-1A	Spring 8 BL-26B2
Space Group	P 1	P 1	P 4_1 2_1 2	P 2 2_1 2_1
Unit cell				
a, b, c (Å)	39.31, 75.68, 76.32	41.52, 76.21, 79.21	82.33, 82.33, 120.27	49.76, 65.53, 125.86
α, β, γ (°)	74.0, 87.2, 84.8	85.6, 84.2, 76.0	90.0, 90.0, 90.0	90.0, 90.0, 90.0
Resolution (Å)	45.7 - 1.60	40.2 - 1.77	67.9 - 1.80	46.3 - 1.90
Wavelength	1.0000	1.0000	1.0000	1.0000
Observations	210,965 (30,305)	290,437 (41,524)	903,851 (74,644)	457,980 (21,635)
Unique reflections	106,948 (15,357)	88,438 (12,726)	39,032 (5,534)	31,867 (1,856)
$R_{merge.}$	0.040 (0.346)	0.087 (0.517)	0.092 (0.468)	0.193 (1.28)
$R_{p.i.m.}$	0.040 (0.346)	0.056 (0.335)	0.019 (0.131)	0.052 (0.379)
$CC_{1/2}$	0.994 (0.810)	0.993 (0.825)	0.999 (0.875)	0.997 (0.677)
$I / \sigma (I)$	8.4 (2.1)	8.4 (2.1)	23.3 (5.6)	14.1 (2.2)
Multiplicity	2.0 (2.0)	3.3 (3.3)	23.2 (13.5)	14.4 (11.7)
Completeness (%)	96.3 (94.7)	96.9 (95.3)	99.9 (99.1)	99.2 (90.2)
**Refinement Statistics**				
Resolution (Å)	45.7 - 1.60	40.2 - 1.77	67.9 - 1.80	46.3 - 1.90
R_{work} / R_{free} (%)	17.2 / 23.5	18.5 / 21.8	17.0 / 21.7	17.9 / 22.9
No. complexes	2	2	1	1
No. atoms				
Protein	6,399	6,365	3,200	3,109
Peptide	144	151	55	51
Other	3	12	1	11
Water	691	737	331	378
B-factor (Å^2)				
Protein	29.3	27.4	23.8	21.3
Peptide	40.9	37.7	25.2	27.7
Others	32.1	32.1	31.6	39.1
Water	33.6	31.4	27.5	25.1
Ramachandran Plot				
Preferred (%)	90.9	90.7	90.5	90.8
Allowed (%)	8.8	9.1	9.2	8.9
Outliers (%)	0.3	0.3	0.3	0.3
RMSD Bond (Å)	0.010	0.012	0.014	0.008
RMSD Angle (°)	1.58	1.73	1.86	1.53
PDB entry code	6LDW	6LDY	6LDX	6LDV
