# Supplementary material for: Structural basis for antigen recognition by methylated lysine–specific antibodies
Source: J Biol Chem. 2020 Dec 17;296:100176. doi: 10.1074/jbc.RA120.015996 (PMC7948472; doi:10.1074/jbc.RA120.015996)
Supplement: Tables S1–S2 [file mmc2.pdf]

F9

VH vs peptide

Hydrogen bonds XML

##	_Structure 1_		_Dist. [Å]_	_Structure 2_	
1	P:THR	12[N]	3.75	H:SER	30[O]
2	P:THR	12[N]	3.01	H:ASN	31[OD1]
3	P:THR	12[OG1]	3.57	H:ASN	31[OD1]
4	P:GLY	8[O]	2.96	H:THR	52[N]
5	P:GLY	8[O]	3.45	H:THR	52[OG1]
6	P:M3L	9[O]	2.89	H:HIS	95[ND1]
7	P:GLY	10[O]	3.01	H:ALA	32[N]
8	P:THR	12[OG1]	3.72	H:ASN	31[ND2]
9	P:TYR	13[O]	2.93	H:TYR	97[N]
10	P:TYR	13[O]	3.33	H:ASN	102[ND2]
11	P:PRO	14[O]	3.71	H:TYR	97[N]
12	P:PRO	14[O]	3.51	H:THR	98[N]
13	P:PRO	14[O]	2.39	H:THR	98[OG1]
14	P:PRO	14[OXT]	2.94	H:ASN	102[ND2]

No disulfide bonds found

No covalent bonds found

No salt bridges found

VL vs peptide

Hydrogen bonds XML

##	_Structure 1_		_Dist. [Å]_	_Structure 2_	
1	P:PRO	14[O]	2.95	L:ARG	48[NH1]
2	P:PRO	14[OXT]	2.83	L:ARG	48[NH2]
3	P:PRO	14[OXT]	3.14	L:ARG	48[NH1]

Salt bridges XML

##	_Structure 1_		_Dist. [Å]_	_Structure 2_	
1	P:PRO	14[O]	3.88	L:ARG	48[NH2]
2	P:PRO	14[O]	2.95	L:ARG	48[NH1]
3	P:PRO	14[OXT]	2.83	L:ARG	48[NH2]
4	P:PRO	14[OXT]	3.14	L:ARG	48[NH1]

No disulfide bonds found

No covalent bonds found

C9

VH vs peptide

Hydrogen bonds XML

Hydrogen bonds XML

##	_Structure 1_	_Dist. [Å]_	_Structure 2_
1	C:GLY 10[N]	3.42	H:SER 97[OG]
2	C:GLY 11[N]	3.09	H:SER 97[OG]
3	C:GLY 8[O]	2.63	H:SER 51[OG]
4	C:GLY 8[O]	3.23	H:THR 53[OG1]

No disulfide bonds found

No covalent bonds found

No salt bridges found

VL vs peptide

Hydrogen bonds XML

##	_Structure 1_	_Dist. [Å]_	_Structure 2_
1	C:GLY 8[N]	2.72	L:ASN 97[OD1]
2	C:GLU 5[O]	3.82	L:ASN 97[ND2]
3	C:LYS 6[O]	3.10	L:ASN 97[ND2]

No disulfide bonds found

No covalent bonds found

No salt bridges found

E6

VH vs peptide

Hydrogen bonds XML

##	_Structure 1_	_Dist. [Å]_	_Structure 2_
1	B:THR 12[N]	3.81	H:ASN 30[O]
2	B:THR 12[N]	2.89	H:ASN 31[OD1]
3	B:THR 12[OG1]	3.22	H:ASN 31[OD1]
4	B:GLY 8[O]	2.85	H:THR 52[N]
5	B:GLY 8[O]	3.45	H:THR 52[OG1]
6	B:GLY 10[O]	2.86	H:ALA 32[N]
7	B:GLY 11[O]	3.84	H:TYR 97[N]
8	B:TYR 13[OH]	2.77	H:GLN 1[NE2]
9	B:TYR 13[O]	2.86	H:TYR 97[N]
10	B:TYR 13[O]	3.39	H:ASN 102[ND2]
11	B:PRO 14[O]	3.21	H:THR 98[N]
12	B:PRO 14[O]	2.52	H:THR 98[OG1]
13	B:PRO 14[O]	3.53	H:TYR 97[N]
14	B:PRO 14[OXT]	2.95	H:ASN 102[ND2]

No disulfide bonds found

No covalent bonds found

No salt bridges found

VL vs peptide

Hydrogen bonds XML

##	_Structure 1_	_Dist. [Å]_	_Structure 2_
1	B:PRO 14[O]	3.18	L:ARG 48[NH1]
2	B:PRO 14[OXT]	2.88	L:ARG 48[NH2]
3	B:PRO 14[OXT]	3.18	L:ARG 48[NH1]

Salt bridges XML

##	_Structure 1_	_Dist. [Å]_	_Structure 2_
1	B:PRO 14[O]	4.00	L:ARG 48[NH2]
2	B:PRO 14[O]	3.18	L:ARG 48[NH1]
3	B:PRO 14[OXT]	2.88	L:ARG 48[NH2]
4	B:PRO 14[OXT]	3.18	L:ARG 48[NH1]

No disulfide bonds found

No covalent bonds found

D6

VH vs peptide

Hydrogen bonds XML

##	_Structure 1_	_Dist. [Å]_	_Structure 2_
1	M:GLY 11[N]	2.86	H:ARG 30[O]
2	M:TYR 13[N]	3.00	H:ASP 98[OD2]
3	M:GLU 5[O]	2.69	H:TYR 53[OH]
4	M:PHE 7[O]	3.12	H:ASN 51[ND2]
5	M:GLY 8[O]	2.94	H:THR 52[N]
6	M:GLY 11[O]	2.70	H:ALA 97[N]

No disulfide bonds found

No covalent bonds found

No salt bridges found

VL vs peptide

Hydrogen bonds XML

##	_Structure 1_	_Dist. [Å]_	_Structure 2_
1	M:TYR 13[OH]	2.93	L:SER 58[N]

No disulfide bonds found

No covalent bonds found

No salt bridges found
