# Supplementary material for: Structural basis for antigen recognition by methylated lysine–specific antibodies
Source: J Biol Chem. 2020 Dec 17;296:100176. doi: 10.1074/jbc.RA120.015996 (PMC7948472; doi:10.1074/jbc.RA120.015996)
Supplement: Supplementary Note 1 [file mmc3.pdf]

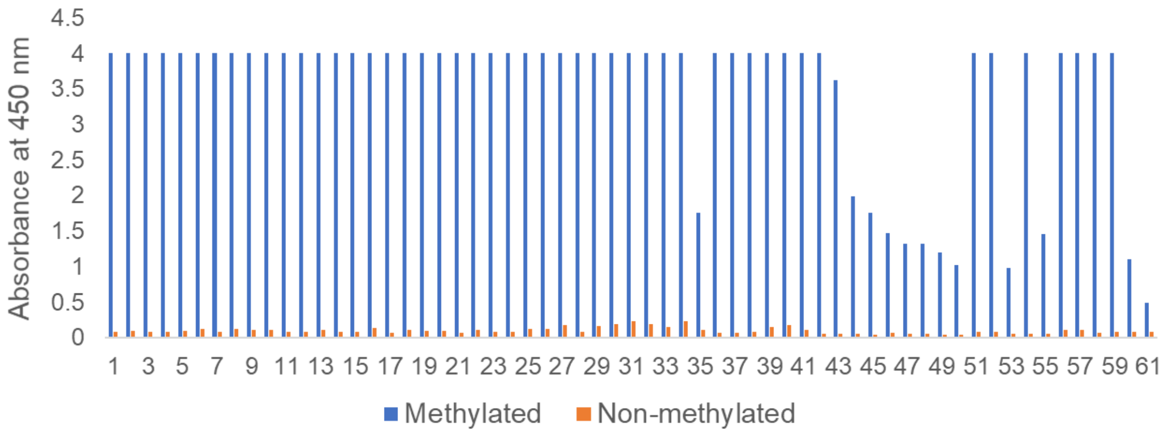

Figure S1: Result of soluble-Fab ELISA. The soluble Fabs were directly expressed from phagemid vectors without subcloning into mammalian expression vectors. The signal responses derived from the binding to methylated and non-methylated peptide antigens were shown with blue and orange bars, respectively.

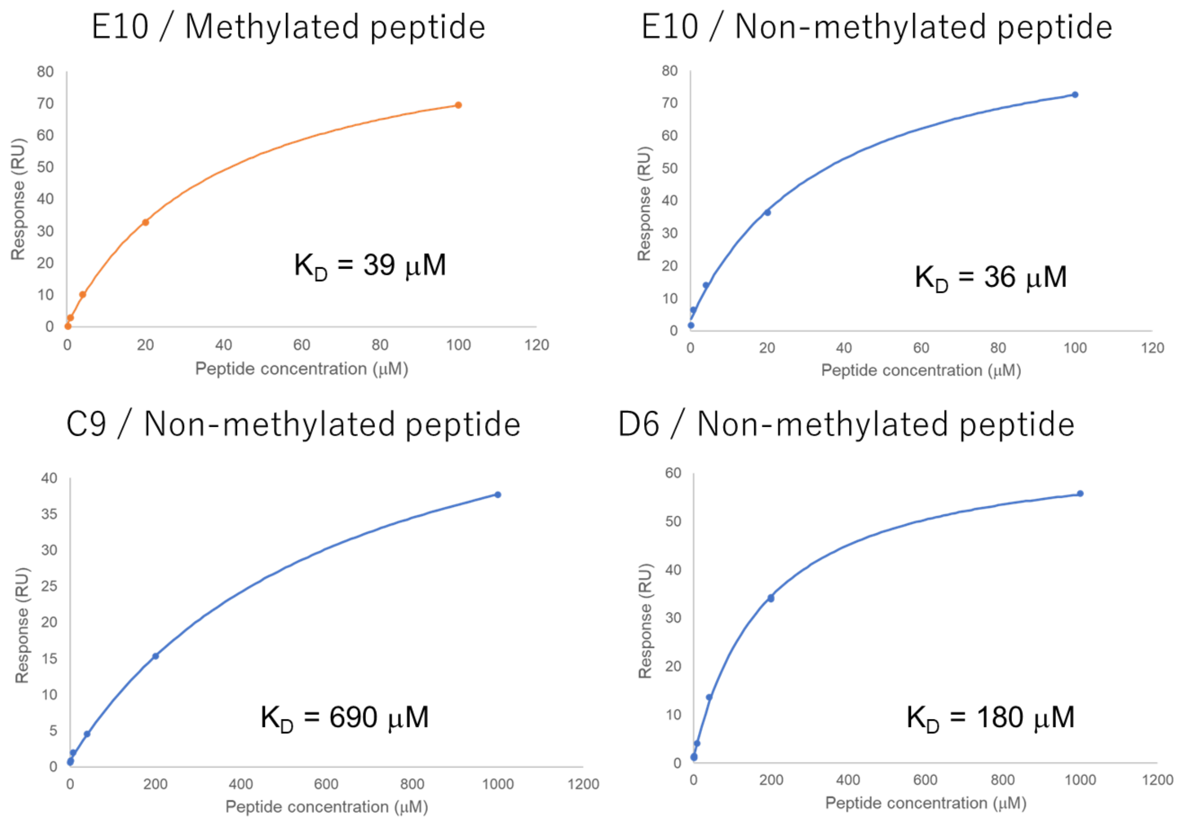

Figure S2: Binding curves from SPR analysis. The binding affinity between peptides and Fabs those kinetics parameters were not determined by global fitting due to low affinity, were determined by steady state analysis.

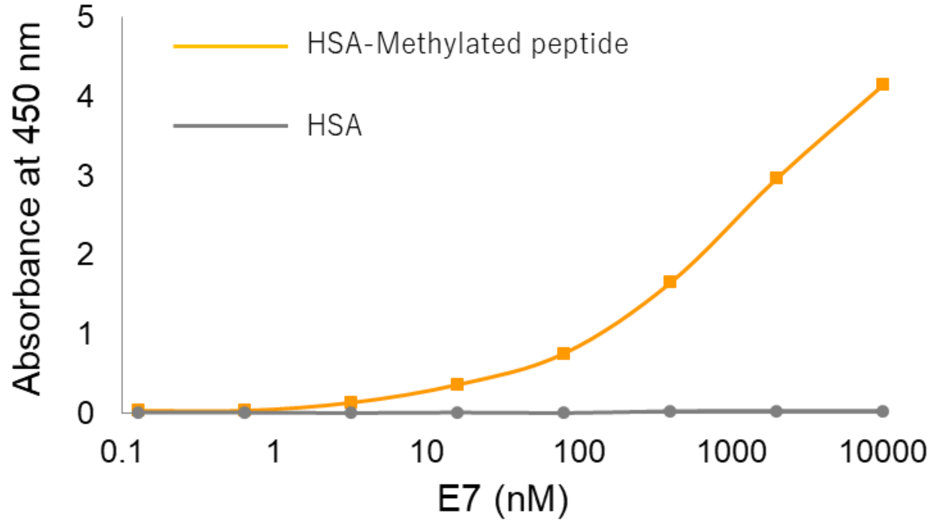

Figure S3: ELISA assay using methylated peptide conjugated with HSA. Higher signal was observed with increasing concentration of Fab, indicating the specific binding of Fab to the conjugated peptide.

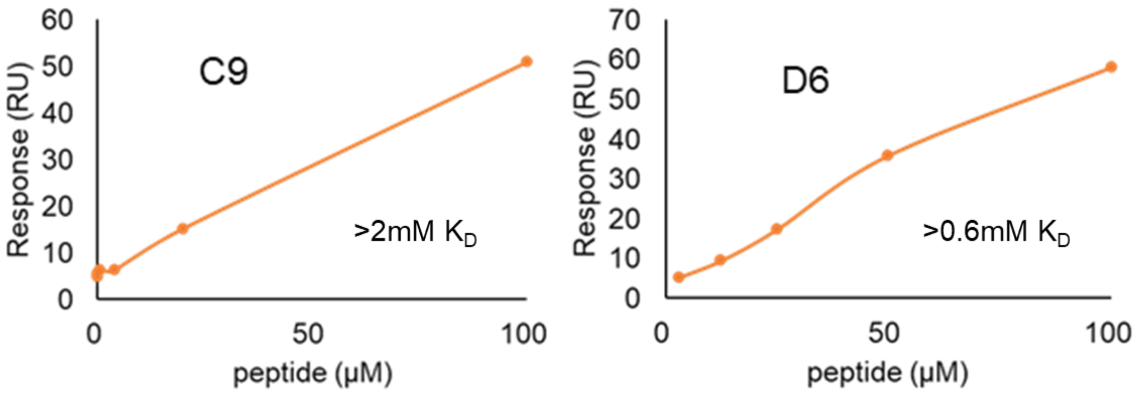

H3K27me3 peptide sequence:
NH_2-QLATKAARK(me3)SAPATG-COOH

antigen peptide sequence:
NH_2-NPIFEKFGK(me3)GGTYP-COOH

Figure S4: Binding curves from SPR analysis using H3K27 trimethylated peptide. Although both C9 and D6 showed binding response to the peptide, the affinity was considerably lower than that to the antigen peptide.

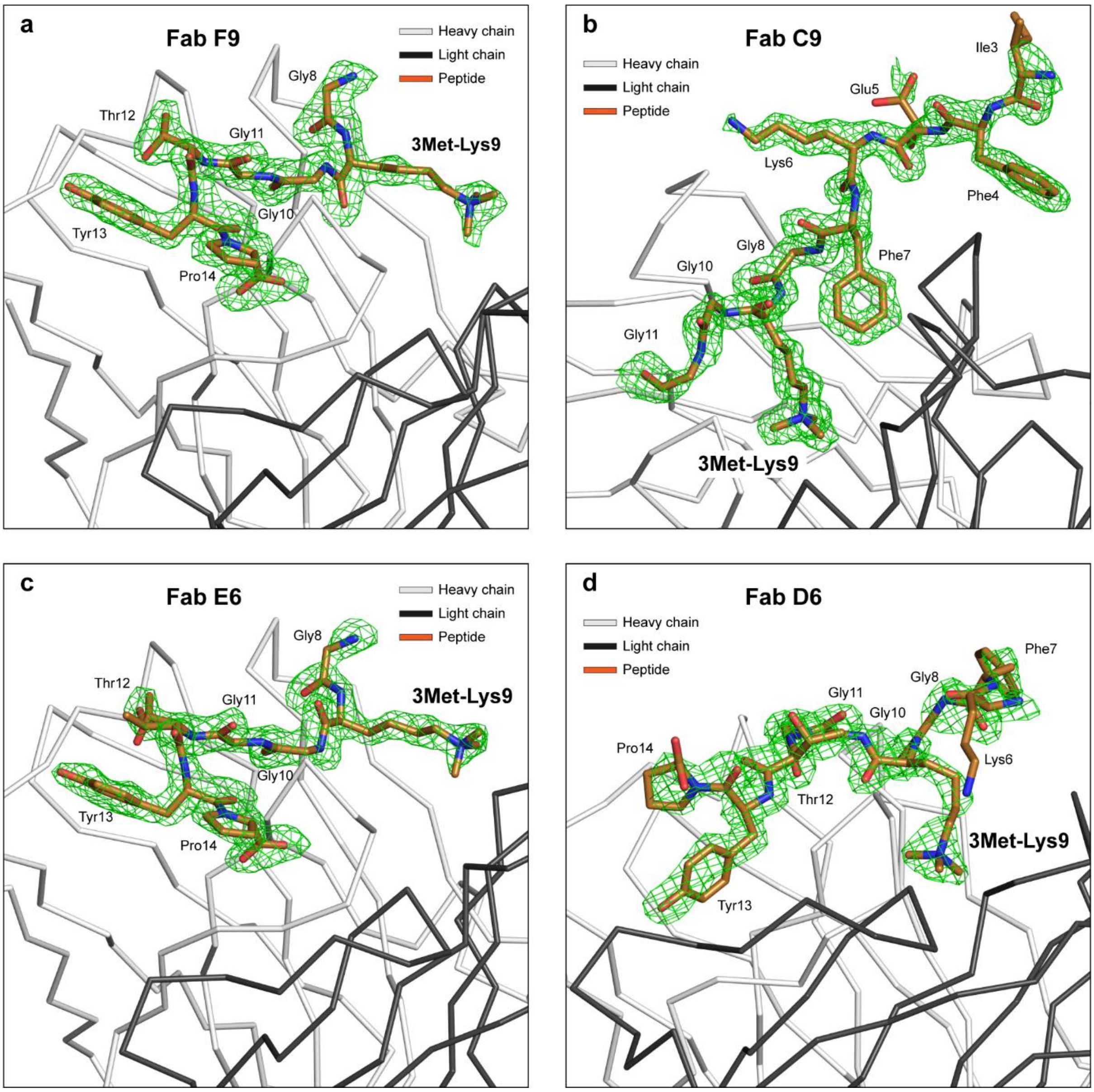

Figure S5. Omit electron density maps. To illustrate the quality of the crystallographic data, omit sigma-A weighted fo-fc electron density maps contoured at a sigma value of 3 (green mesh) were calculated for **(a)** Fab F9, **(b)** Fab C9, **(c)** Fab E6 and **(d)** Fab C9. In all panels the heavy chain and light chain are shown as light and dark gray ribbon, respectively. The peptide is depicted with sticks, where dark orange, blue an red correspond to carbon, nitrogen and oxygen atoms.

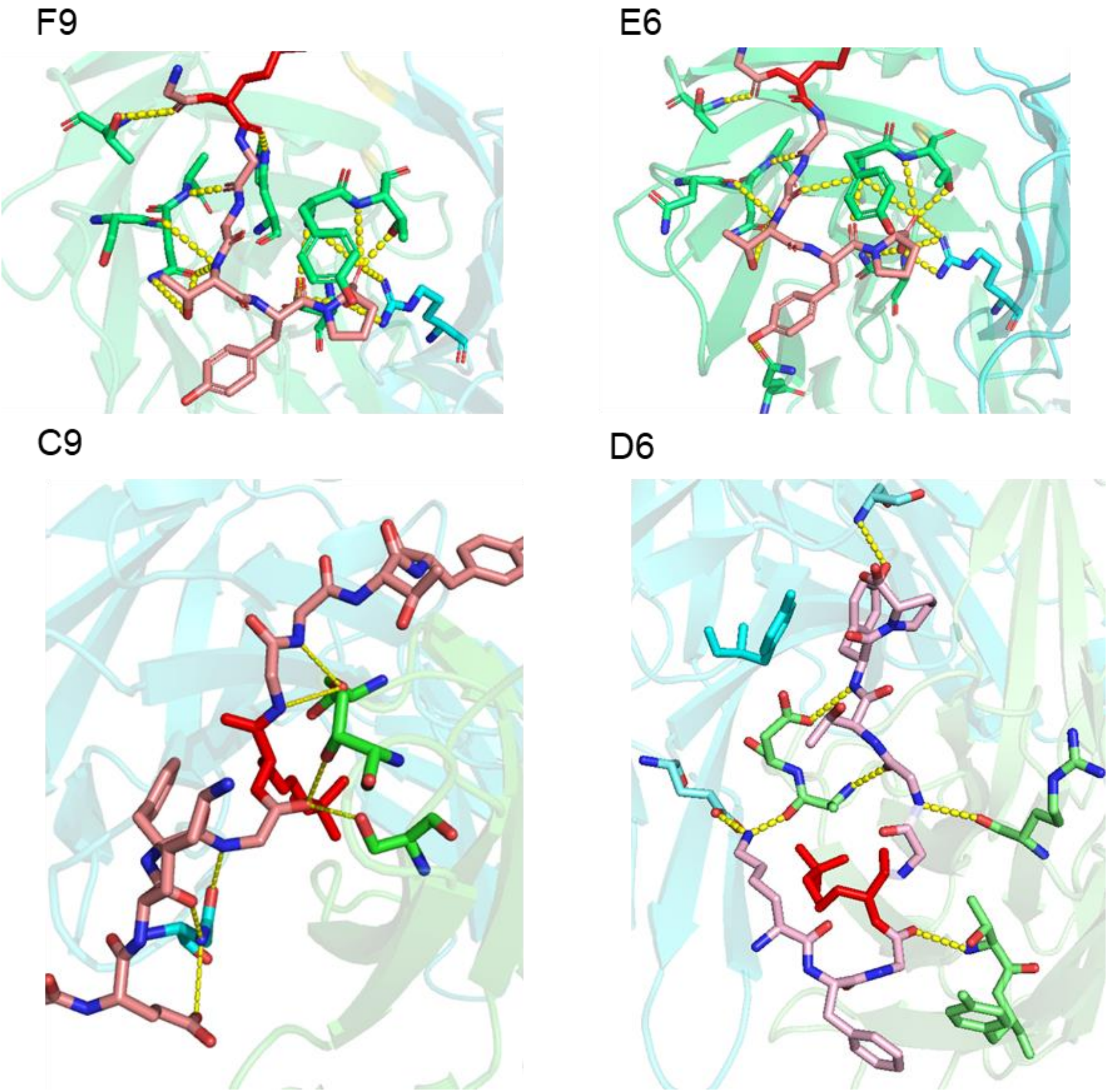

Figure S6 Hydrogen bonds between the peptide and Fabs. Hydrogen bonds between Fab and the methylated peptide. The bonds are shown in yellow colored dot lines.

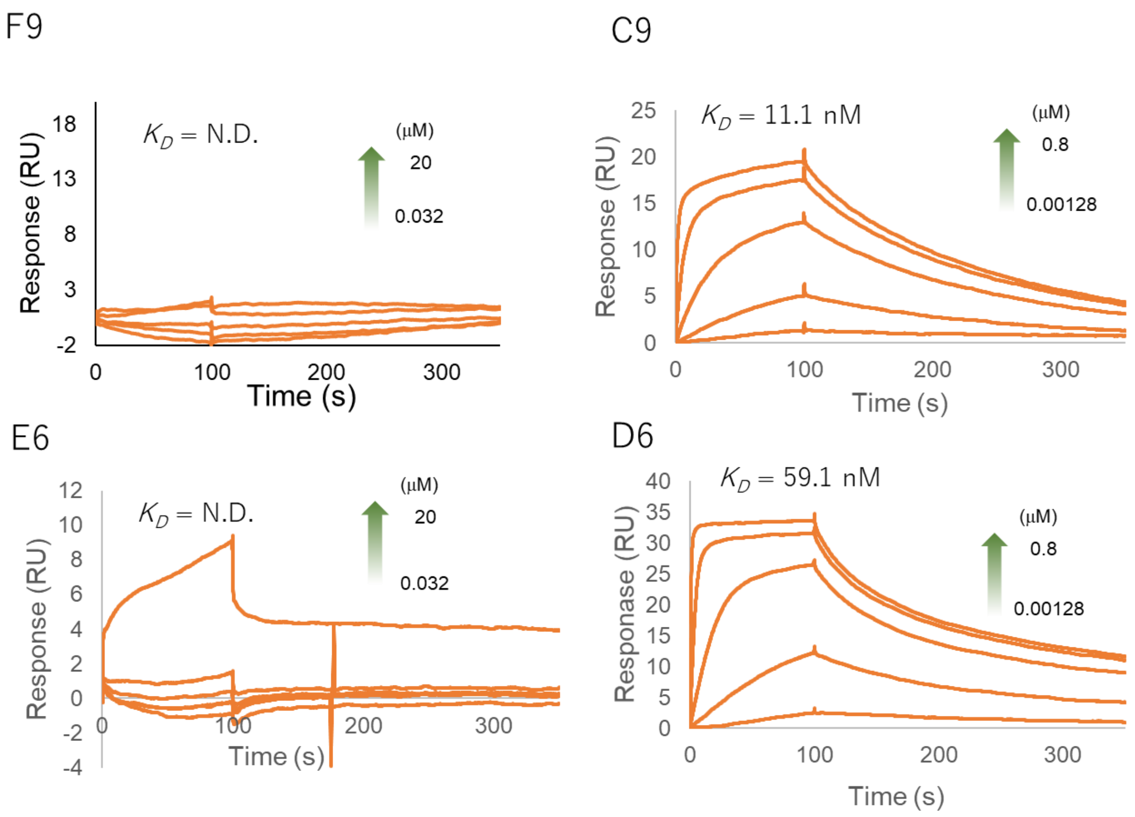

Figure S7: SPR analysis using C-terminal amidated peptide. Affinity of the antibodies toward C-terminal amidated peptide was investigated by SPR. The binding of F9 and E6 to the peptide was nearly abolished by the amidation.

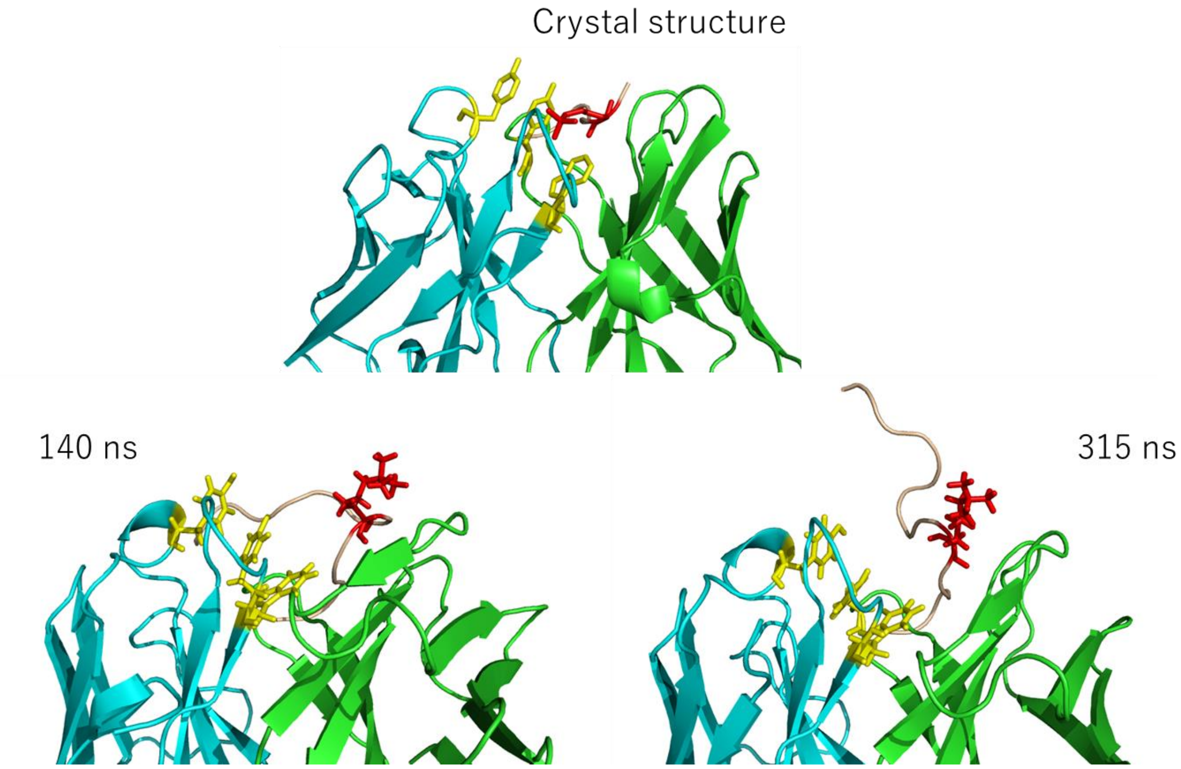

Figure S8: Crystal structures of F9– peptide complex at each time point. The structures with collapsed aromatic cage were observed at above time points.

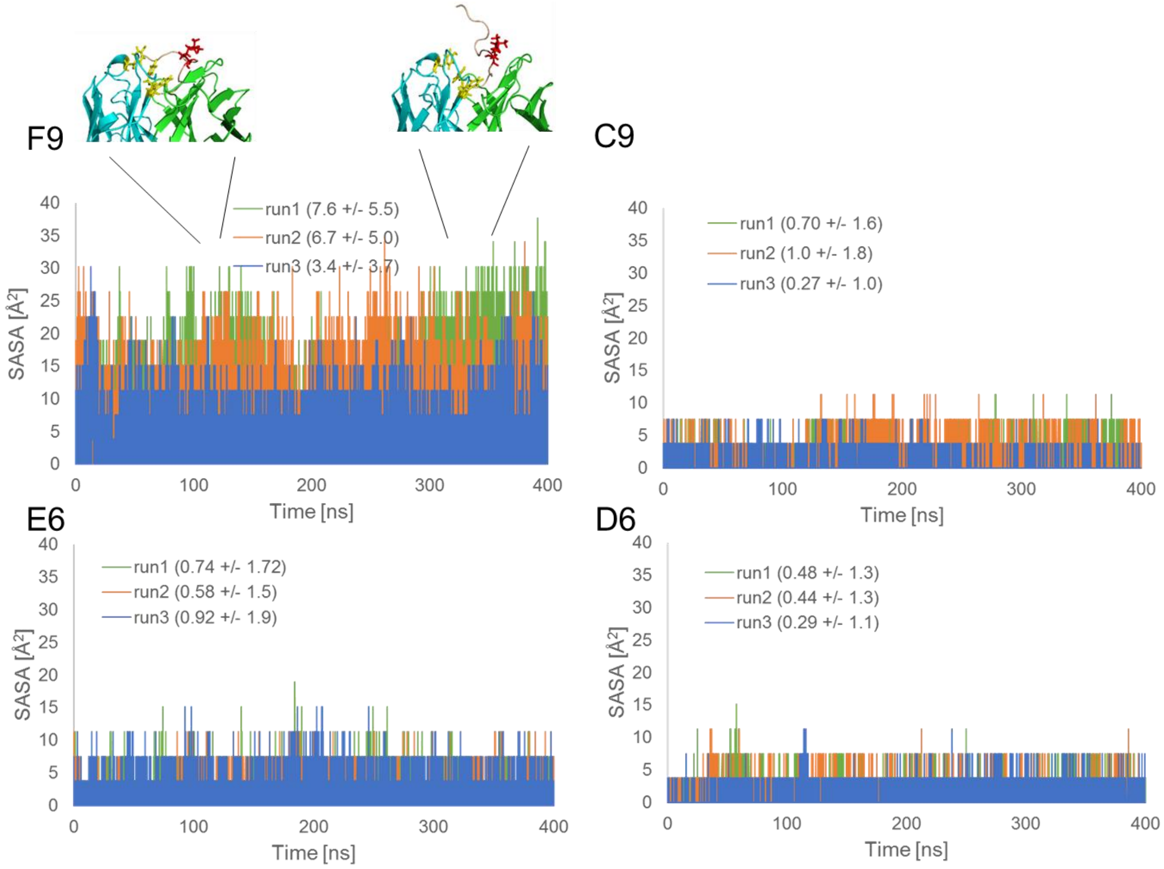

Figure S9: ASA values of the methylated lysine residue in each Fab – peptide complex. ASA values for F9 were significantly larger than that for other antibodies throughout the simulations.

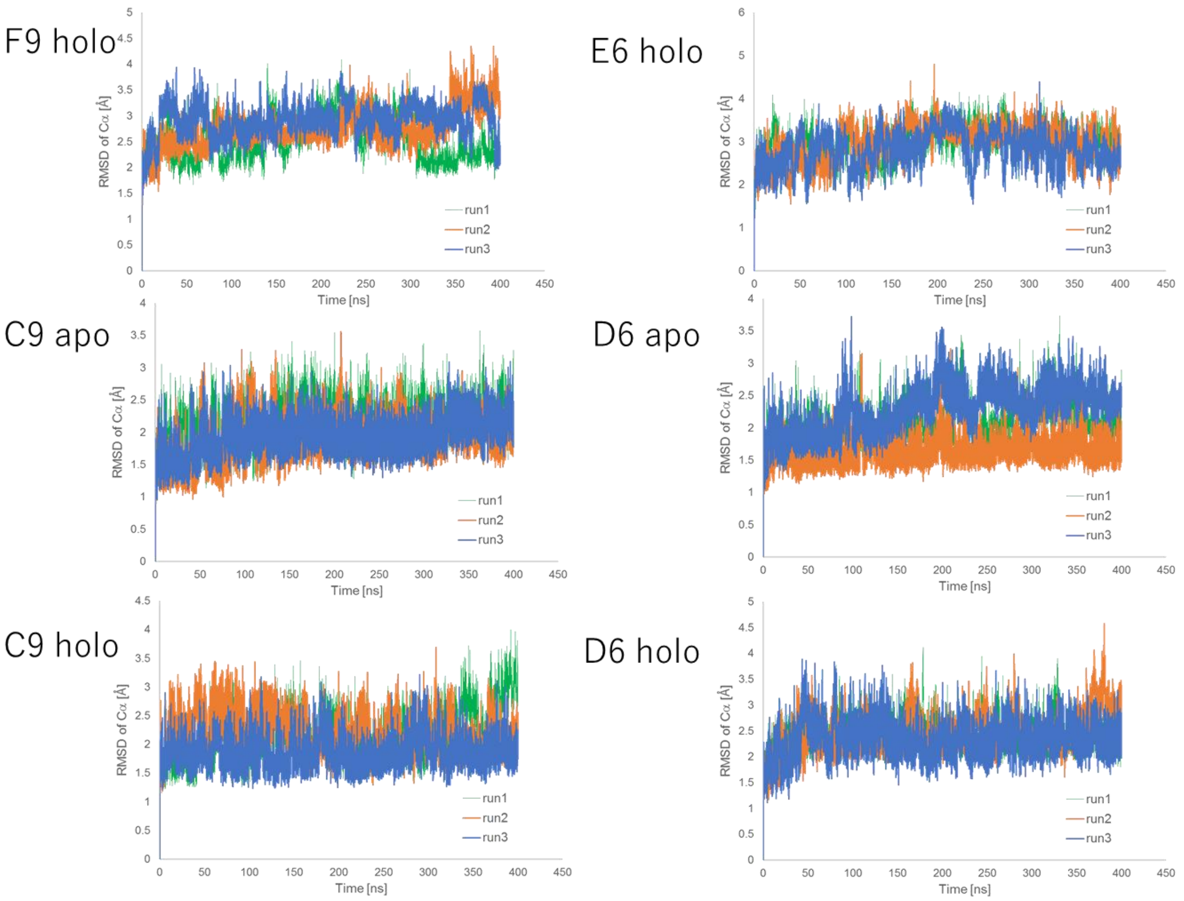

Figure S10: RMSD values of total Ca atoms in each Fab – peptide complex. RMSD values for each simulation were analyzed to validate the simulation. The RMSD values for all the simulations became stable after 10 ns.
